# Plastic Operation for Restoration of the Sphincter Ani, with Report of a Case

**Published:** 1902-10

**Authors:** 


### Plastic Operation for Restoration of the Sphincter Ani, With Report of a Case.

Under this heading Chas. H. Chetworth, in *New York Medical Record,* describes a very ingenious method for restoration of this necessary muscle. The *modus operandi* in the case cited consisted in raising a semi-circular flap, extending from the coccyx to either side as far as a line drawn between the tuberosities of the ischium, thus exposing the lower part of the rectum and the edges of the gluteii muscles. Thin strips one-fourth inch wide by one-sixteenth inch thick were dissected on each side from these muscles, attachments being left above, the strips were then made to encircle the gut, the right hand fibres going to the left side and the left hand fibres to the right, being fastened together in front of the anus with catgut. The operation proved to be eminently successful and complete control of the bowel contents resulted. C. R. M.

